# Prospective Register Of patients undergoing repeated OFfice and Ambulatory Blood Pressure Monitoring (PROOF-ABPM): protocol for an observational cohort study

**DOI:** 10.1136/bmjopen-2016-012607

**Published:** 2016-10-31

**Authors:** James P Sheppard, Una Martin, Paramjit Gill, Richard Stevens, Richard J McManus

**Affiliations:** 1 Nuffield Department of Primary Care Health Sciences, University of Oxford, Oxford, UK; 2 School of Clinical and Experimental Medicine, University of Birmingham, Birmingham, UK; 3 Institute of Applied Health Research, University of Birmingham, Birmingham, UK

**Keywords:** PRIMARY CARE, Secondary Care, PREVENTIVE MEDICINE, Diagnostics

## Abstract

**Introduction:**

The diagnosis and management of hypertension depends on accurate measurement of blood pressure (BP) in order to target antihypertensive treatment appropriately. Most BP measurements take place in a clinic setting, but it has long been recognised that readings taken out-of-office (via home or ambulatory monitoring) estimate true underlying BP more accurately. Recent studies have shown that the change in clinic BP over multiple readings is a significant predictor of the difference between clinic and out-of-office BP. Used in combination with patient characteristics, this change has been shown to accurately predict a patient's out-of-office BP level. The present study proposes to collect real-life BP data to prospectively validate this new prediction tool in routine clinical practice.

**Methods and analysis:**

A prospective, multicentre observational cohort design will be used, recruiting patients from primary and secondary care. All patients attending participating centres for ambulatory BP monitoring will be eligible to participate. Anonymised clinical data will be collected from all eligible patients, who will be invited to give informed consent to permit identifiable data to be collected for data linkage to external outcome registries. Descriptive statistics will be used to calculate the sensitivity, specificity, positive and negative predictive values of the out-of-office BP prediction tool. Area under the receiver operator characteristic curve statistics will be used to examine model performance.

**Ethics and dissemination:**

Ethical approval for this study has been obtained from the National Research. Ethics Service Committee South Central—Oxford A (reference; 15/SC/0184), and site-specific R&D approval has been acquired from the relevant NHS trusts. All findings will be presented at relevant conferences and published in peer-reviewed journals, on the study website and disseminated in lay and social media where appropriate.

**Strengths and limitations of this study**

- This study will be the first prospective register of patients undergoing routine clinic and ambulatory blood pressure (BP) monitoring in a UK setting.
- It will be powered to examine the accuracy of a new out-of-office BP prediction tool in routine clinic practice.
- Despite the broad inclusion criteria for patients in the study, the registry is unlikely to capture many patients with normal clinic BP readings, since these patients are not routinely referred for ambulatory BP monitoring in routine clinical practice.
- The registry is therefore unlikely to capture many patients with masked hypertension, although it will capture those displaying a large masked effect.

## Introduction

High blood pressure (BP) (hypertension) is an important risk factor for cardiovascular disease,^1^ a significant cause of morbidity and mortality worldwide. The diagnosis and management of hypertension depends on accurate measurement of BP in order to target antihypertensive treatment at those with the most to gain.^2^ The majority of BP measurements take place in a clinic setting; however, it has long been recognised that readings taken over a 24-hour period estimate true underlying BP more accurately because multiple readings can be averaged and it correlates better with a range of cardiovascular outcomes compared with clinic BP.^3–5^ Indeed, clinic BP often misclassifies this true underlying BP which can lead to incorrect diagnosis and management.^6^ ^7^

**Figure 1:**
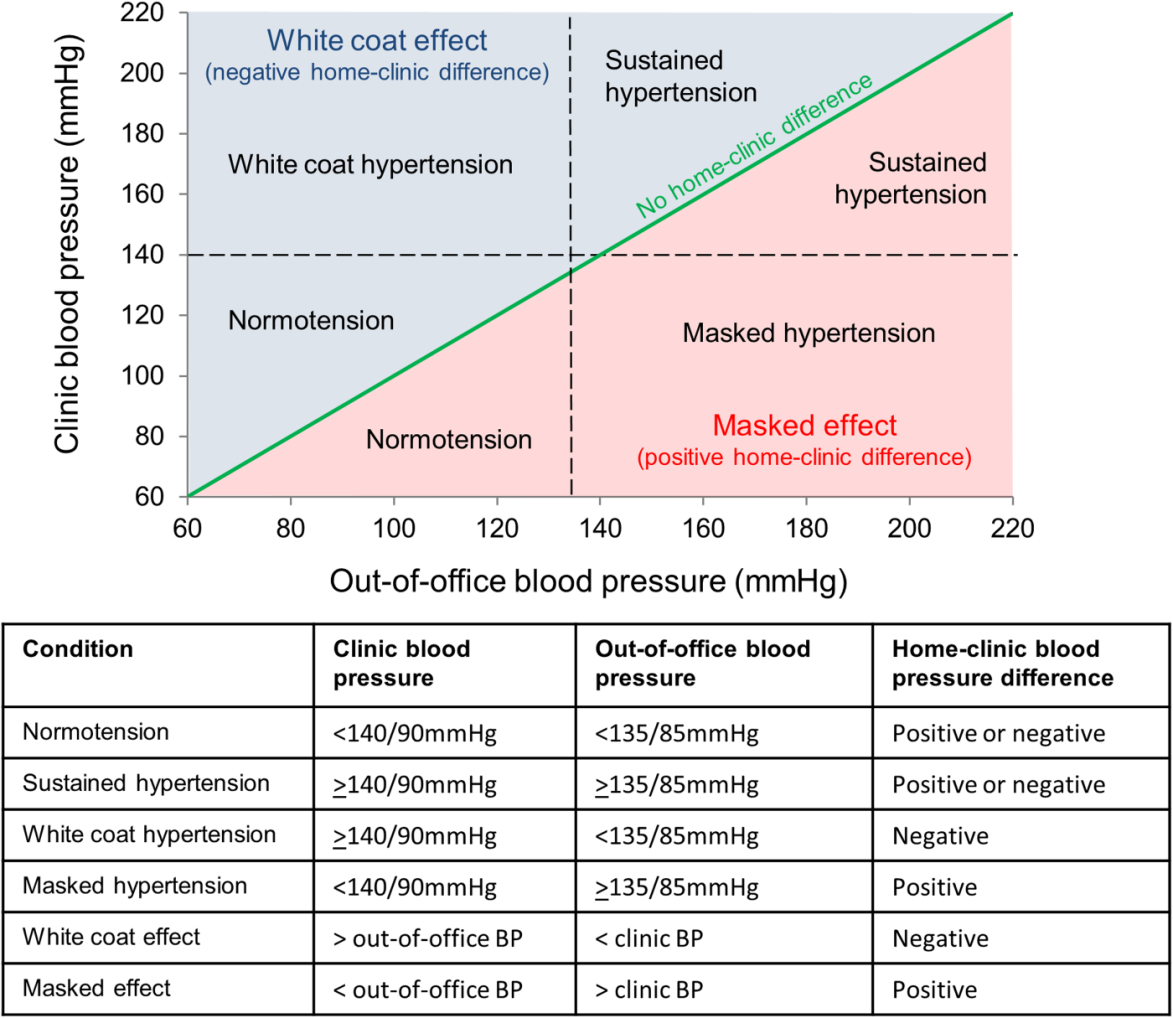
Definitions of normotension, hypertension and the home-clinic blood pressure (BP) difference. BP, blood pressure; out-of-office BP may be defined by home or daytime ambulatory BP measurements. Individuals with a white coat effect (negative home-clinic difference) may be normotensive, hypertensive or white coat hypertensive. Those with a masked effect (positive home-clinic difference) may be normotensive, hypertensive or masked hypertensive. Those with an out-of-office >135/85 mm Hg (hypertension) may be masked or sustained hypertensives.

Depending on the direction of the error, such deviations are defined as a ‘white coat’ or ‘masked’ effect (figure 1).^8^ ^9^ Patients with a significant white coat effect have high clinic BP and a lower daytime or 24-hour ambulatory BP and are at risk of overtreatment.^8^ Conversely, patients with a significant masked effect have high BPs with daytime or 24-hour ambulatory monitoring but lower corresponding clinic BPs. These patients are often underdiagnosed and potentially undertreated,^9^ thereby leading to increased risk of target organ damage^10^ and cardiovascular mortality.^11^ ^12^ White coat and masked hypertension are terms used to describe occasions where patients displaying such deviations have clinic and ambulatory BP readings on opposite sides of the diagnostic threshold for hypertension (figure 1).

In the UK, the National Institute for Health and Clinical Excellence (NICE) now recommend out-of-office measurement (ambulatory or home monitoring) if BP is raised in the clinic to confirm a diagnosis of hypertension.^13^ While this method of diagnosis is considered cost-effective due to a reduction in misdiagnosis caused by white coat hypertension,^2^ it still results in patients with true underlying hypertension identified by clinic BP readings being sent for arguably unnecessary out-of-office monitoring and will not capture those patients with masked hypertension.

Work by some of the authors^14^ has shown that the change in clinic BP over multiple readings is a significant predictor of the home-clinic BP difference: a decrease in clinic BP across multiple readings is associated with lower BP at home and *vice versa.* We have subsequently confirmed this effect using data from previous trials^15–18^ and shown that, in combination with patient characteristics, this change can be used to accurately predict a patient's out-of-office BP level.^19^ Used as a triaging tool for ambulatory BP monitoring, the PROOF-BP prediction tool^19^ permits detection of those patients with a possible white coat or masked effect on the basis of data available in a routine primary care clinic. The tool could also be used to rule out the white coat effect in resistant hypertensives and assist in the monitoring of BP target attainment following treatment titration which might be more relevant for secondary care populations.

It is well known that BP measurements made under controlled conditions in a research setting are not necessarily comparable to those made by a physician in routine clinical practice.^20–22^ Differences can occur from the use of inadequate or uncalibrated devices,^23–25^ suboptimal measurement techniques^26–28^ or rounding bias (such as terminal digit preference).^29^ ^30^ Thus, a prediction model shown to be accurate in a research setting is not guaranteed to be as precise in routine clinical practice. The present study proposes to collect sufficient data from routine practice to prospectively validate the PROOF-BP prediction tool and better understand the relationship between BPs measured in different settings and how they are related to cardiovascular disease risk.

This will be achieved by setting up a prospective register of patients attending routine clinical practice for office and ambulatory BP monitoring. The register will comprise of patient characteristics and clinical data and be complementary to existing BP monitoring registries,^31^ ^32^ many of which are based in specialist hypertension clinics around the world, but unique in its consideration of multiple clinic BP readings taken in variety of healthcare settings. Indeed, although such data are routinely collected in clinical practice, there are no current databases which capture this information for research purposes. Even linked anonymised databases such as the Clinical Practice Research Datalink (CPRD) do not collect all the information that will be captured within this database since multiple individual clinic and ambulatory BP readings (from a single visit or monitoring period) are rarely captured on general practice or hospital computer systems.

## Methods

### Study design

This study will use a prospective, multicentre observational cohort design, recruiting patients from primary care and secondary care and pharmacies where possible. The study will run from 1 May 2015 until 31 December 2017, or until a sufficient number of patients have been enrolled to enable the planned statistical analyses to be undertaken. Subsequent follow-up of patient admissions to hospital and mortality will continue after this date.

### Study participants and setting

This study will include consecutive patients attending participating centres in primary or secondary care (or pharmacies) for routine BP screening. This will include patients identified with raised BP during routine checks in primary care, those referred to Secondary Care with suspected hypertension, newly treated hypertension, resistant hypertension, secondary hypertension or other specialist conditions and those referred (by their general practitioner) to local pharmacies for ambulatory BP monitoring. Eligible patients will be invited to give informed consent to allow identifiable data to be collected for data linkage to external registries: the Office for National Statistics (ONS) mortality and Hospital Episodes Statistics (HES) databases via NHS Digital's Data Linkage and Extract Service. The register will be web-based to permit access from a variety of healthcare settings.

### Inclusion criteria

This study will use broad inclusion criteria to capture all patients attending clinical practice for routine ambulatory BP monitoring:

- male and female participants,
- age 18 years or above,
- attending clinical practice for routine ambulatory BP monitoring.

### Exclusion criteria

Participants may not enter the study if any of the following apply:

- aged under 18 years old,
- lack of availability of basic clinical information,
- multiple clinic BP readings (obtained on at least three occasions within the same visit) not recorded,
- ambulatory BP monitor not worn as instructed (ie, invalid measurements taken).

### Recruitment and informed consent

**Figure 2:**
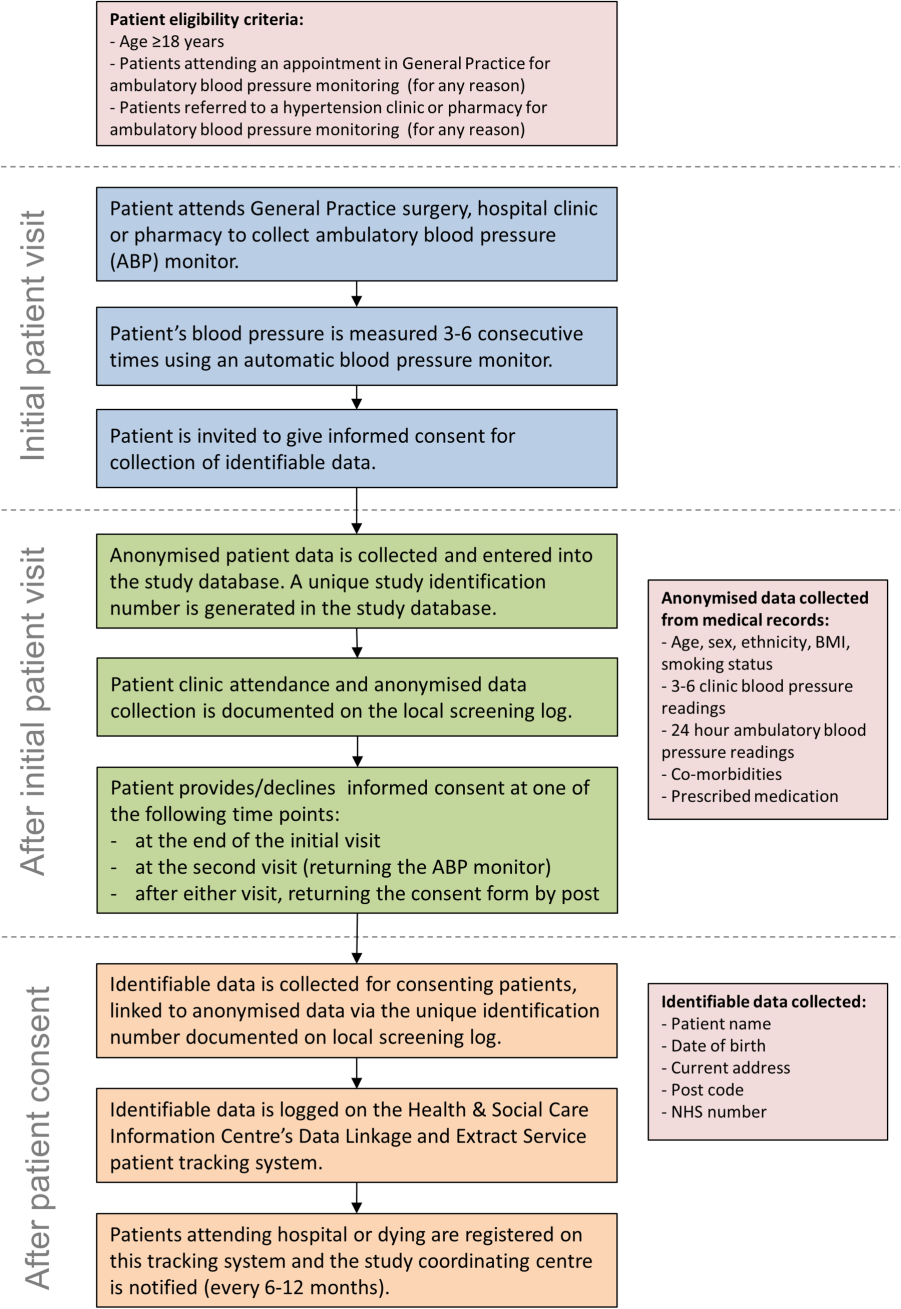
Patient recruitment and data collection.

Practices and pharmacies will be approached via the local NIHR Clinical Research Network, hospital sites will be contacted by the research team directly. Sites which are certified as having undergone training in Good Clinical Practice (GCP) will be targeted in the first instance. Patients attending each study site will be screened opportunistically, that is, potential patient records will not be screened prior to invitation to participate, but rather those attending routine clinical practice for ambulatory BP monitoring will be approached by a member of the clinical care team and invited to give informed consent. Anonymised clinical data will be collected for all patients approached and identifiable patient data will be collected in those giving informed consent (figure 2).

The participant information sheet and informed consent form will be presented to potential participants by the consulting healthcare team. The study participant will personally sign and date the latest approved version of the informed consent form before any study-specific identifiable data collection is carried out. The participant will be allowed as much time as they wish to consider the information, and the opportunity to question the investigator, their general practitioner or other independent parties to decide whether they will participate in the study. The process of taking informed consent will use the methods established in the DESCARTE and Cough Complications Cohort (3C) studies.^33^ ^34^ That is, eligible patients will be able to provide informed consent in one of three ways:

1. During clinic attendance, patients will be ask by participating staff if they would be willing to consider giving consent for identifiable data to be collected for data linkage purposes. Patients will be given an information sheet and consent form and if willing to do so, complete it during the same clinic.
2. Those wishing more time to consider participation will be asked to take the information home and, if willing to participate, return a completed consent form during their next visit when they return the ambulatory BP monitor.
3. Those forgetting to provide consent at either visit may return a signed consent form to the participating centre by post.

All investigators taking informed consent at participating centres will be expected to have undergone training in GCP. Where this is not the case, training will be offered via the NIHR Clinical Research Network. Each participant will retain a copy of the signed informed consent form and the original will be retained at the study site. A copy of the signed form will be scanned and uploaded onto the study database.

### Anonymised data collection

Anonymised clinical data from all patients eligible for the study will be collected and entered onto the study database by participating staff at each data collection site. Individual patient consent is not required for anonymised data collection and will not be sought, as is common in routine clinical audits and anonymised observational cohort studies,^35^ ^36^ although those wishing to opt out will be able to if they wish. All sites will be offered an automated BP monitoring device (Omron M10-IT or equivalent) to assist with the collection of multiple clinic BP readings, but can choose to continue using their standard monitor, so long as at least three readings are taken and recorded. The Omron M10-IT device automatically takes three clinic readings at 30 s to 1 min intervals and calculates an average. The individual readings and the averaged BP can be viewed on the monitor after measurement. Current guidelines in the UK^13^ and abroad^21^ ^37^ recommend that 2–3 clinic readings are taken when screening for hypertension and thus collection of data for the minimum data set required in the present study will not constitute a deviation from usual care (although documentation of these individual readings may incur additional time for which participating centres will be reimbursed, where appropriate). Instructions will be provided for clinic and ambulatory BP monitoring (based on clinical guidelines),^13^ but no formal procedure will be put in place for checking if measurement protocols have been adhered to; such flexibility will be allowed to reflect true clinical practice. Clinic readings taken at the time of referral for ambulatory BP monitoring or at monitor fitting will be deemed acceptable for inclusion in the study, but no limit on the period of time between the two will be specified. Where available, data from repeated clinic/ambulatory measurements in the same patient will be collected and used to assess the sensitivity of the tool used with repeated measurements.

A minimum data set will be required for all eligible patients and include patient characteristics and clinical data related to BP measurements and cardiovascular disease risk (table 1). Where data are routinely collected and available, additional information relating to specific cardiovascular risk factors will be collected to permit subgroup analyses by risk group (table 1).

**Table 1:** Identifiable and anonymised data variables

	Anonymised clinical data	
Identifiable patient data	Minimum data set	Additional variables (if available)*
Patient name	Age	Waist circumference
Date of birth	Sex	Alcohol consumption
Current address	Ethnicity	Right and left arm clinic blood pressure
Post code	Smoking status	History of left ventricular hypertrophy
NHS number	Height	Sodium
	Weight	Potassium
	3–6 clinic blood pressure readings	Calcium
	Daytime ABP	Total cholesterol
	Night-time ABP	HDL cholesterol
	24 ABP	Triglycerides
	Number of readings	HbA1c
	Reason for monitoring	Plasma renin levels
	Diagnosis of hypertension	Creatinine
	History of hypertension	eGFR
	History of myocardial infarction	Albumin:creatinine ratio
	History of stroke	Urinalysis
	History of heart failure	Thyroid-stimulating hormone
	History of diabetes	Free Thyroxine (T4)
	History of chronic kidney disease	Albumin
	History of atrial fibrillation	Alanine transaminase (ALT)
	Antihypertensive prescription	Aspartate aminotransferase (AST)
	Statin prescription	Alkaline phosphatase (ALP)
	Antiplatelet prescription	Total bilirubin
		γ-Glutamyltransferase

*Blood analyses are only routinely conducted in Secondary Care. Only measurements taken during the baseline visit will be deemed acceptable for inclusion.

ABP, ambulatory blood pressure; eGFR, estimated glomerular filtration rate; HbA1c, glycated haemoglobin; HDL, high-density lipoprotein; NHS, National Health Service.

### Identifiable data collection

Eligible patients will be invited to give informed consent to allow identifiable data to be collected for data linkage to external registries. The prespecified identifiable data to be collected are detailed in table 1. These data will be used to link clinical data collected at baseline to outcome data via the NHS Digital's Data Linkage and Extract Service. This service tracks patient events via the Hospital Episode Statistics and the Office for National Statistics death register, using NHS numbers and other identifiable information and will allow ascertainment of all hospital admissions and/or death status following enrolment into the study.

### Study database

Each data collection site will store their data locally using standard clinical systems and upload a copy of these data to a secure central database at the study coordinating centre (University of Oxford). Local staff will be trained to upload data and automated checks will be used to ensure data entry errors are kept to a minimum. Data will be entered into two separate study databases, one for anonymised data and one for identifiable data. A unique study identifier will automatically be generated for every patient entered onto the anonymised study database and this will be entered onto the database of patient identifiers for those patients giving informed consent (permitting linkage of identifiable and anonymised data). Double data entry will be employed for entry of the unique study identifier onto the database of patient identifiers to ensure accuracy. Both study databases will include secure login for staff at participating sites and facilities for manual data entry, upload of ambulatory BP monitoring data and consent forms (contained in .csv and .pdf files).

### Data protection and storage

Identifiable data will be stored at each data collection site in accordance with data protection guidelines and NHS policy, and on the secure study database hosted by the coordinating institution. Data access will be limited to specific members of the research team (trained in data protection policy), including the principle investigator (as study guarantor), data manager and database programmer. All data used in analyses and published outputs will be anonymised. Applications for data sharing with researchers from other research organisations will be reviewed by the registry steering committee and decisions on access will be subject to satisfactory review of a study protocol. All data and study documentation will be stored for subsequent scientific validation and audit as required. Data will be archived following completion of the study in line with GCP guidelines and documents will be retained for a minimum of 5 years after publication in line with the University Code of Practice for Research.

## Statistical analysis

### Outcomes

The primary outcome of this study will be to define the accuracy of the PROOF-BP prediction tool^19^ in terms of the proportion of true-positive, true-negative, false-positive and false-negative results in a typical population attending routine clinical practice with suspected or diagnosed hypertension. Secondary outcomes will be examined where sufficient data are available and include assessment of model performance in different subgroups—age (young vs old), cardiovascular disease risk (high vs low risk according to previous history and risk scores using data where available), those with chronic kidney disease, diabetes and across healthcare settings: primary care, secondary care and pharmacy settings.

In the longer term, linked data from the registry will be used to examine how BP data collected at baseline predict long-term clinical outcomes (eg, hospital admission with myocardial infarction/stroke and mortality). It is anticipated that the registry will become a useful source of data permitting further investigations into BP monitoring by a variety of means and cardiovascular disease risk factor data linked to cardiovascular disease morbidity and mortality in routine clinical practice.

### Data analysis

A detailed analysis plan will be agreed to by the study steering committee prior conducting any analyses. Briefly, data collected from the registry will be used to prospectively validate the PROOF-BP prediction tool. Descriptive statistics will be used to define the number of patients classed as true positives (sustained hypertensives), false positives (white coat hypertensives), true negatives (normotensives) and false negatives (masked hypertensives) (figure 1). These will be used to calculate the sensitivity, specificity, positive predictive value and negative predictive value of the PROOF-BP prediction tool. Area under the receiver operator characteristic (AUROC) curve statistics will be used to examine the prediction model performance.

χ^2^ statistics will be used to compare the classification of patients' hypertensive status according to the prediction model and existing strategies^13^ ^37^ for the diagnosis and management of hypertension. An improvement in patient classification of >10% or reduction in the usage of out-of-office monitoring of >20% will be deemed as successful validation. These thresholds were chosen because they were deemed to represent a clinically significant improvement in diagnostic accuracy which might be expected to change clinical practice. Where model validation is found to be unsuccessful, recalibration will be explored.

Other secondary outcomes will be examined with χ^2^ statistics comparing the classification of patients' hypertensive status across subgroups (by setting, age group, sex, cardiovascular disease risk status, comorbid conditions and treatment status). Sensitivity analyses will exam the accuracy of the PROOF-BP prediction tool using 24 hour BP to define the home-clinic BP difference, where it is collected. Linked data will be used to examine the association between the ‘adjusted clinic blood pressure’ (estimated from the PROOF-BP prediction model) and clinical outcomes (all-cause mortality, cardiovascular mortality, hospital admission with myocardial infarction or stroke) using the Cox proportional hazards models.

### Sample size

Based on the initial validation phase of the PROOF-BP prediction model, conducted using data from previous studies,^19^ accrual of data from up to 1000 patients would allow for estimation of hypertensive status with an accuracy of ±1–3%. This assumes 71% of patients will be classed as true positives, 24% as true negatives, 3% as false positives and 2% as false negatives. In a population of 1000 patients, it would be possible to estimate these rates with the following 95% CIs: true positive 71% (68% to 74%), true negative 24% (21% to 27%), false positive 3% (2% to 4%) and false negative 2% (1% to 3%) (table 2). If these point estimates were to differ, or indeed a slightly lower number of patients were enrolled, the impact on the accuracy of the study results would be minimal (table 2). The sample size of up to 1000 patients has been specified to ensure that the prespecified subgroup analysis can be undertaken and be adequately powered.

**Table 2:** Sample size estimates and the impact on the precision of the study results, taking into account uncertainty about the expected prevalence of normotension and white coat hypertension

		Sustained hypertensive (true positive)		Normotensive (true negative)		White coat hypertensive (false positive)		Masked hypertensive (false negative)	
Scenario	Sample size	Point estimate	Predicted CI	Point estimate	Predicted CI	Point estimate	Predicted CI	Point estimate	Predicted CI
Prevalences observed in the original PROOF-BP study^19^	1000	71%	68% to 74%	24%	21% to 27%	3%	2% to 4%	2%	1% to 3%
	800	71%	68% to 74%	24%	21% to 27%	3%	2% to 4%	2%	1% to 3%
Prevalence of normotension decreases by 50%	1000	83.5%	81% to 86%	12%	10% to 14%	3.5%	2% to 5%	1%	0.5% to 2%
	800	83.5%	81% to 86%	12%	10% to 14%	3.5%	2% to 5%	1%	0.5% to 2%

Prevalence of normotension decreases by 75%	1000	89.5%	87% to 91%	6%	5% to 8%	4%	3% to 5%	0.5%	0% to 1%
	800	89.5%	87% to 92%	6%	4% to 8%	4%	3% to 6%	0.5%	0% to 1%

Prevalence of white coat hypertension increases by 50%	1000	69.5%	67% to 72%	24%	21% to 27%	4.5%	3% to 6%	2%	1% to 3%
	800	69.5%	66% to 73%	24%	21% to 27%	4.5%	3% to 6%	2%	1% to 3%

Prevalence of white coat hypertension increases by 100%	1000	68%	65% to 71%	24%	21% to 27%	6%	5% to 8%	2%	1% to 3%
	800	68%	65% to 71%	24%	21% to 27%	6%	4% to 8%	2%	1% to 3%

PROOF-BP, predicting out-of-office blood pressure in the clinic.

Approximately 182 patients would be required in each subgroup to examine the secondary outcomes proposed in the proposed study. This is based on a likelihood ratio test of two proportions detecting a 10% difference in the classification of hypertensive status between two subgroup populations (primary care vs secondary care, primary care vs pharmacies, older vs younger patients, high-risk vs low-risk patients) with a significance level of 0.05 and 90% power. Assuming correct classification of 95% of patients in one group and 85% in the other, ∼364 patients (182 in each group) would be required to demonstrate this significant difference. Thus, our recruitment target of 1000 patients should be sufficient to answer primary and secondary outcomes, provided recruitment is appropriately distributed across clinic settings and patient characteristic subgroups.

Recruitment of the sample size above should be achievable with recruitment of up to 10 primary care/pharmacy sites (each recruiting up to 50 patients) and one secondary care site (recruiting up to 500 patients). With patient attendance for routine BP monitoring at individual sites likely to be significantly higher, these targets are eminently achievable.

## Ethics and dissemination

Ethical approval for this study has been obtained from the National Research Ethics Service Committee South Central—Oxford A (reference; 15/SC/0184), and site-specific R&D approval has been acquired from the relevant NHS trusts.

All findings will be presented at relevant conferences and published in peer-reviewed journals, on the study website and disseminated in lay and social media where appropriate. The investigators will be involved in reviewing drafts of the manuscripts, abstracts, press releases and any other publications arising from the study. The authors will acknowledge that the study was funded by the Medical Research Council. Authorship will be determined in accordance with the ICMJE guidelines and other contributors will be acknowledged.

Relevant results will be made available for the next iterations of the NICE Hypertension guidelines and other relevant national guidelines. It is anticipated that these will support better patient-centred management plans for the diagnosis and management of hypertension and cardiovascular disease risk.

## Discussion

This protocol describes the first prospective register of patients undergoing routine clinic and ambulatory BP monitoring in a UK setting. This is important, since the UK is leading the way in the promotion of ambulatory BP monitoring for the diagnosis and management of hypertension.^13^ Thus, the proposed register is likely to contain a broader range of patients attending routine practice than other registries based in specialist hypertension clinics around the world,^31^ ^32^ and unique in its consideration of multiple clinic BP readings taken in variety of healthcare settings.

Despite the broad inclusion criteria for patients to be included in the study, it is unlikely to capture many patients with normal clinic BP readings, since these patients are not routinely referred for ambulatory BP monitoring in routine clinical practice. Thus, the registry is unlikely to capture many patients with masked hypertension (although it will capture those displaying a large masked effect) and further work will be required to validate the PROOF-BP prediction tool^19^ in routine practice in this particular population. The register will however permit the prospective validation of this tool using data from real-life clinical settings, and allow the accuracy of existing BP monitoring strategies to be examined in routine clinical practice. It is hoped that this work will improve the diagnosis and management of hypertension in primary and secondary care, allowing better targeting of treatment at those patients with the most to gain.
